# Structural Insights into Broadly Reactive SARS-CoV-2 Antibodies for Vaccine Design

**DOI:** 10.1063/4.0001158

**Published:** 2025-10-27

**Authors:** Morgan E Abernathy, Christopher O Barnes

**Affiliations:** Stanford University, Stanford, CA, USA

## Abstract

Broadly reactive monoclonal antibodies (mAbs) elicited by SARS-CoV-2 vaccination or infection can provide valuable insight into the types of antibody immune responses that would be required for a future, cross-reactive coronavirus vaccine. Here, we study two classes of mAbs: those that bind the receptor-binding domain (RBD) and cross-react with SARS-CoV-2 variants, and those that bind the stem helix and exhibit even broader cross-reactivity across the betacoronavirus genus. Using both single-particle cryo-EM and X-ray crystallography, we investigated these antibodies in complex with their antigens, which allows for analysis of important contacts. By examining these interactions in the context of the antibodies’ maturation pathways and common viral mutations, we can gain insight into how some mAbs evolve to retain activity against emerging variants. These findings highlight the structural and evolutionary features that may be valuable to elicit in future betacoronavirus vaccine design.

